# Development and exploration of the content validity of a patient-reported outcome measure to evaluate the impact of migraine- the migraine physical function impact diary (MPFID)

**DOI:** 10.1186/s12955-017-0799-1

**Published:** 2017-11-17

**Authors:** Asha Hareendran, Sally Mannix, Anne Skalicky, Martha Bayliss, Andrew Blumenfeld, Dawn C. Buse, Pooja R. Desai, Brian G. Ortmeier, Sandhya Sapra

**Affiliations:** 1 Evidera, Metro Building, 6th Floor, 1 Butterwick, London, W6 8DL UK; 2 Evidera, Bethesda, MD USA; 3 Optum, Lincoln, RI USA; 4 Headache Center of Southern California, Encinitas, CA USA; 5 Department of Neurology, Albert Einstein College of Medicine and Montefiore Medical Center, Bronx, NY USA; 6 Amgen Inc., Thousand Oaks, CA USA

**Keywords:** Migraine, Content validity, Cognitive interview, Development, Functioning, Instrument, MPFID, Pro, Diary, Headache, Item generation, Disability, Impact

## Abstract

**Background:**

Adults with migraine experience substantial reductions in quality of life during and in-between migraine attacks. Clinical and regulatory guidelines encourage the inclusion of patient reported outcomes for the evaluation of benefits of interventions for migraine.

**Methods:**

The conceptual framework and items for a new patient-reported outcome (PRO) instrument, the Migraine Physical Function Impact Diary (MPFID), were developed using scientific methods recommended to ensure content validity of PRO instruments. The MPFID was developed to measure the impact of migraine on physical functioning based on themes raised in concept elicitation (CE) interviews (conducted previously) with adults with migraine. Cognitive interviews were conducted with adults with migraine to further explore content validity. The instrument was modified following an interim analysis of a first round of cognitive interviews, to assess comprehensiveness and clarity of items, instructions, and response options. Refinements were subsequently tested in additional cognitive interviews.

**Results:**

The conceptual framework included impacts on physical functioning experienced by most adults with migraine and deemed clinically relevant for measuring the outcome of an intervention for migraine. Concepts in the framework included the impact of migraine on physical impairments (acts) and ability to complete day-to-day activities and perform everyday activities (tasks). MPFID items were generated to evaluate functioning over the past 24 h and to collect data daily, to capture experiences on days with migraine as well as the days in-between migraines. Items asked about needing to rest or lie down; ability to get out of bed, stand up, bend over, walk, perform household chores, do tasks outside the home, keep routines or schedules, get ready for the day, do activities that require concentration or clear thinking; difficulty moving head and body, doing activities requiring physical effort; avoiding interacting with others. Initial modifications based on the first round of cognitive interviews (*n* = 8) included clarifying instructions, updating three items to enhance specificity and clarity, and revising one item to include gender-neutral language. The second round of interviews (*n* = 9) confirmed acceptability of revisions and supported content validity.

**Conclusions:**

The results provide qualitative evidence supporting the content validity of the MPFID for evaluating outcomes of interventions for migraine.

**Electronic supplementary material:**

The online version of this article (10.1186/s12955-017-0799-1) contains supplementary material, which is available to authorized users.

## Background

Adults with migraine experience recurrent headaches lasting four to 72 h, often with unilateral, pulsating, moderate or severe headache, frequently aggravated by routine physical activity [1]. In addition to these symptoms, people with migraine experience significant headache-related disability including substantial reductions in their quality of life in comparison with the general population [2–7], absenteeism from work or school [8, 9], and reduced productivity due to migraine when at work or school [10].

A systematic review of literature to identify psychosocial difficulties associated with migraine found that the most frequently reported difficulties included - reduced vitality and fatigue, emotional problems, pain, difficulties at work, general physical and mental health, social functioning and global disability [11]. Leonardi et al., exploring impacts of migraine using the broader framework of World Health Organization’s (WHO) International Classification of Functioning, Disability and Health (ICF) found that in addition to the symptoms of migraine and impact on daily activities, migraine patients reported difficulties with changing and moving body positions [12].

More recently, concept elicitation interviews conducted to the point of saturation, with adults with migraine also showed that adults with migraine experienced impacts of migraine on physical social and emotional functioning [13]. The ***impacts of migraine on physical functioning were*** deemed to be the most appropriate outcome to evaluate the immediate benefits of interventions that prevented migraines. Impacts were reported on ‘acts’ (things that an individual can do independent of context or purpose) and tasks (things people do in daily life in a specific context, with purpose). Acts and tasks were the key components of the ‘activity component’ of the ICF as clarified by Badley [14].

Impacts on functioning are experienced before and between attacks because of prodromal symptoms preceding their attack or the need to avoid situations in their daily lives that could precipitate or aggravate their condition (eg, bright lights, loud noises, physical activity [4]. Capturing the day-to-day variability in patients’ experiences of migraine to understand both ictal and inter-ictal impacts of migraine is therefore essential for a comprehensive understanding of the patients experience [3, 4, 13]. Prophylactic treatments that aim to prevent migraine attacks or reduce the number of migraine days should therefore also aim to improve day-to-day functioning in these patients. Guidelines for the management and evaluation of treatments for migraine emphasize the importance of collecting data about the patient reported impact of migraine on functioning [15–17].

Numerous generic patient reported outcome (PRO) instruments used in migraine studies [5] have proven to be useful for comparing the burden of migraine to population norms or other disease benchmarks; however, more precise measurement can be achieved with disease-specific PRO instruments for evaluating the benefits of treatment as they are often more clinically relevant and responsive to treatment [18]. The US Food and Drug Administration (FDA) guidance for PRO instruments to support labeling claims recommends that, PRO instruments used to determine treatment efficacy must reflect the patient experience; their content should be identified based on qualitative concept elicitation studies with the target sample, ensuring content validity [19, 20].

As part of a review of disease specific instruments evaluating the impact of migraine [13]it was determined that a few PRO instruments commonly used in migraine studies do contain items about the impact of migraine on physical functioning tasks (eg, the Headache Impact Test™ (HIT-6™) [21], Migraine Specific Quality of life questionnaire (MSQ) [22], and the Migraine Disability Assessment (MIDAS) questionnaire [23]) they did not capture impacts on acts (eg, difficulty moving the body). These instruments were developed before the FDA PRO guidance was published in 2009 and gaps were identified in terms of qualitative evidence supporting their content validity as required by the FDA PRO guidance.

This manuscript describes the development and qualitative testing of a new PRO instrument- The Migraine Physical Function Impact Diary (MPFID) using best practice methods suggested for ensuring content validity of PRO instruments to support treatment benefit claims [19, 20].

## Methods

**Fig. 1:**
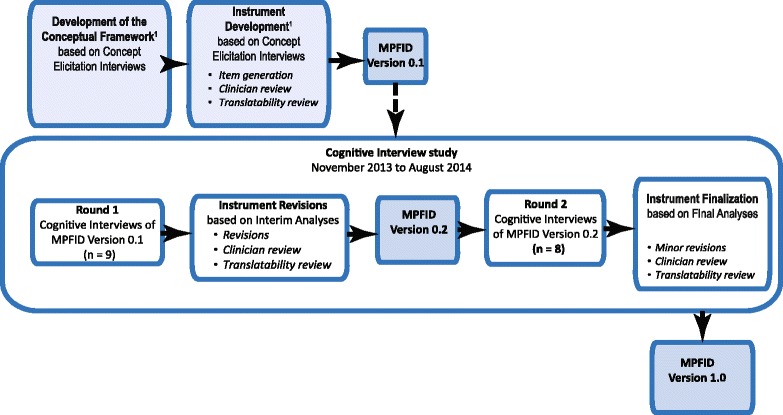
Overview of the Flow of the Project. The MPFID was developed using an iterative process primarily based on results of qualitative interviews with adults with migraine. ^1^Not reported in this manuscript. See Mannix et al. [13]

The MPFID was developed using an iterative process. The instrument was developed to reflect the experiences of adults with migraine from concept elicitation research conducted to the point of saturation [13]. A conceptual framework for measuring the concept of interest—***impact of migraine on physical functioning—***was derived from analyses of the transcripts from these concept elicitation interviews. The conceptual framework was developed and refined in consultation with migraine experts to ensure clinical relevance for use in evaluating interventions for migraine. This conceptual framework guided the generation of the content for the initial version of the MPFID. The initial version of the MPFID was refined based on feedback from – cognitive interview with adults with migraine and by migraine clinicians and translatability experts as described in Fig. 1 and summarized below.

### Development and refinement of the conceptual framework (CF)

The process used to develop the conceptual framework for the MPFID started with a qualitative analysis of the transcripts from the CE study [13]. A coding dictionary was developed based on symptoms of migraine and impacts of migraine. Participant statements (eg, words and phrases) were labeled with codes by trained coders. Coded data was systematically reviewed and summarized to document the most relevant and prominent migraine impact concepts for inclusion in the CF. The coding procedure has been described previously [13].

Initial versions of the CF were discussed and revised by the MPFID development team and migraine experts (two neurologists with expertise in treating migraine patients, a clinical psychologist with expertise in headache and pain management, and an instrument development expert with expertise in migraine-specific PRO tools). The conceptual framework was revised based on feedback from migraine experts to help ensure inclusion of dimensions salient to patients with migraine and deemed clinically relevant for measuring the outcomes of interventions on the impact of migraine on physical functioning (the context of use).

### Instrument development

#### Item generation

To accurately reflect patients’ experiences with migraine symptoms, coded data were reviewed and items were generated based on patients’ words used to describe the concepts in the CF. Item writing guidelines as described by Streiner and Norman [24] were followed for item generation (eg, creation of questions, establishment of the recall period, selection of proposed response choices, and grouping of items into relevant domains). A pool of previously validated questionnaire items identified by the PRO development team was also reviewed to inform item construction, while taking care to ensure that constructed items reflected the terminology used by participants in the concept elicitation interviews. Response choices were drafted and aligned with the item stems by using participants’ descriptions of the variability in migraine impact.

#### Expert reviews

Early drafts of the MPFID were reviewed by the four migraine experts at two points during instrument development (Fig. 1). Recommended changes were evaluated in conjunction with results from the concept elicitation interviews before they were implemented.

Since the MPFID was intended for global use, after draft items were developed, an expert in PRO translations conducted an assessment of ‘lexibility’ (grade level and readability), followed by an assessment of translatability. This expert provided feedback about words or phrases that could be structurally or culturally problematic when translated into different languages. In addition, the original English version of the measure was evaluated for neutrality or “universal” English, so that the measure could be appropriate for use in the United States, United Kingdom, Australia, and New Zealand. Translatability assessments were conducted at three time points: before the first round of cognitive interviews, after revisions to be tested in the second round, and on the final version (Fig. 1).

### Cognitive interviews to test initial version of the MPFID

The MPFID version 0.1 was tested in adults with migraine to a) further evaluate the content of the instrument and b) to assess participants’ understanding of items, instructions, and response options, and to evaluate whether their interpretation matched the intent of the developers in the context of its use; Two rounds of cognitive interviews were conducted with a clinic-based convenience sample of adults with migraine. After the first round of interviews, results of an interim analysis of qualitative data, migraine expert review and translatability review led to revisions to the MPFID, which was then tested in a second round of interviews.

A sample size comparable to published suggestions of 5–15 subjects per round was targeted [20]. The number of participants needed to reach saturation for item review and assessment of comprehension was estimated to be close to 15 participants total; with 6–8 participants interviewed per round. The point of saturation was an assessment made by the project team, with support from the data, to determine whether any new issues were emerging during the second round of cognitive interviews [20].

Adults (18 to 60 years of age) with migraine, with or without aura, were recruited through two clinical sites in the United States using a standard screening script and a standard set of inclusion/exclusion criteria aimed to match eligibility criteria for studies of interventions for migraine prophylaxis (Additional file 1: Table S1). Participants had to have a history of migraine (International Headache Society [IHS] International Classification of Headache Disorders 2nd Edition [ICHD-II] [25]) for at least 12 months. *Note: the study protocol was finalized in early 2013 ahead of publication of the 3rd Edition*. Key exclusion criteria included migraine onset older than 50 years of age, cognitive impairment preventing participation in the interview, and participation in the prior concept elicitation interview study [13].

All study procedures were approved by a central Institutional Review Board (IRB), and participants provided written informed consent prior to data collection procedures. Additional descriptive information was captured using study-specific case report forms.

The one-on-one in-person interviews were conducted by 2 researchers with interviewing experience who were trained by senior researchers (SM and AS) with a combined 15 years of experience in conducting qualitative interviews. Training sessions included review of cognitive interviewing best practice methods, review of interview guide, mock interviews, and feedback on first interviews conducted. Interviews were conducted in-person in clinical sites and interviewers greeted the participants, explained the objective of the study, reviewed the informed consent form and answered any study questions before initiating the interview.

As the intention was to use the MPFID on an electronic hand-held device in future studies, the MPFID content was formatted to simulate screens on an electronic platform for testing. The MPFID was presented to and completed by participants with instructions and items with one item per page. After reviewing and responding to all items, participants were interviewed about the relevance of each item and were asked targeted questions to assess comprehensiveness of content, including questions about the instructions, items and response options. A semi-structured cognitive interview guide including tables to capture interview notes was used to guide the cognitive interviews.

All interviews were audio-recorded and transcribed. Interview transcripts were imported into ATLAS.ti (version 7.1) qualitative data analysis software [26]. Transcripts were coded using a coding dictionary developed from the cognitive interview guide, interviewer notes, and team discussion. Participant statements (e.g., words and phrases) were labeled with codes based on responses to questions about comprehension of the instructions, recall period, item content, and responses options to capture this information for analyses. This approach allowed for in-depth evaluation of quotes within each code.

Results of an interim analysis based on interviewer notes after the first round of interviews were used to modify the instructions, items, and response options. Final analyses were conducted using data from the second round of interviews and all transcripts were coded by applying the coding dictionary as described above. An item development tracking matrix captured evidence to support each item and to track revisions made to items following cognitive testing and translatability assessments. To characterize and quantify participants’ headache-related health status, participants completed the Headache Impact Test™ (HIT-6™), a six item PRO measuring the impact of migraine in terms of pain, social functioning, role functioning, vitality, cognitive functioning, and psychological distress [21].

## Results

### Development and refinement of the conceptual framework

The conceptual framework was developed to include impacts frequently experienced by at least 50% of the participants in the concept elicitation interviews [13] and included impacts on ‘acts’ (ability to move parts of their body and ambulation), as well as their ability to do many daily activities(tasks) [13]. Migraine experts highlighted the need to avoid concepts that were not necessarily an impact of migraine but potentially a result of co-morbid conditions (eg, a pervasive feeling of lack of energy), which may result from many aspects of patients’ physical and mental health and may not be directly attributed to a migraine (and therefore not relevant to evaluate the benefit of a prophylactic treatment for migraine). The experts also suggested the inclusion of an overall impact on everyday activity concept to evaluate the patients’ overall perspective of the impact of migraine, because such an item could be useful to help monitor outcomes of interventions aimed at preventing migraines in clinical practice.

**Fig. 2:**
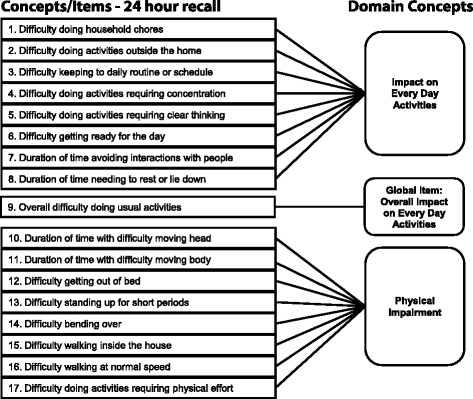
Conceptual Framework of Version 1.0 of the MPFID. The conceptual framework comprised 17 concepts/items in 3 domain concepts representing impact on every day activities, overall impact on every day activities, and physical impairment

The conceptual framework is shown in Fig. 2.

These dimensions of the conceptual framework were used to guide the development of the initial versions of the MPFID.

### Instrument development

The item stems, instructions, and response options of the 17 items of MPFID version 0.1 were developed to reflect the dimensions in the conceptual framework and were based on language used by participants during qualitative interviews [13] to ensure the appropriateness, relevance, understanding, and clarity of included items.

The instructions described the intent of the MPFID as assessing the respondent’s ability to function and to understand how a migraine affects the respondent’s ability to perform day-to-day activities and directed the respondent to answer all questions by selecting the one option that best described her experience.

The instrument was developed for completion every day to capture the day-to-day variability of the impacts of migraine on physical functioning as identified in the concept elicitation interviews, which demonstrated that the impact of migraines varied on a daily basis, during and between migraine episodes, and also varied with the severity of migraine [13]. A 24-h recall period was chosen to avoid recall bias.

The response options were selected to reflect the experiences described by participants in the concept elicitation interviews [13]. When describing their experiences with migraines and the effects on physical functioning, participants provided detailed accounts of how they often “powered through” the difficulties and how long they experienced difficulties with activities (eg, duration of difficulty moving their head), or the level of difficulty in performing certain activities. Participants with frequent and severe migraines reported great difficulty with physical functioning, to the extent that they were sometimes unable to perform many everyday activities. To capture these dimensions salient to participants and relevant for daily assessment, a Likert-type response scale using a 5-point ordinal scale was developed. The level of impact on physical function was measured in the terms of “without any difficulty” to “unable to do” or “not difficult” to “extremely difficult” (eg, for the household chores item); response options of “none of the time” to “all of the time” were used to collect data about concepts that participants described in terms of duration of difficulty during a period of time (eg, difficulty moving the head item) [13].

The initial draft MPFID was reviewed by the migraine experts and the translation expert for revisions prior to testing in cognitive interviews. The migraine experts suggested revisions to items to evaluate specific concepts (eg, activities inside and outside the home, ability to keep a schedule, interactions with others) and proposed considerations for gender neutral terminology in items such as getting ready for the day (eg, the item on ability “to make yourself presentable” that included examples of brushing hair, shaving, and applying make-up, was revised to clarify the concept as “getting ready for the day” and the examples were removed to ensure that the item was gender neutral). Expert insights into respondent burden (eg, appropriate number of items and responses, frequency of administration) were also considered. The translation expert recommended changes to certain sentence structures (eg, “how much did you have to limit your social activities because of a migraine?” instead of “how much did you have to limit your participation in social activities because of a migraine”), items (eg, “outside your home” instead of “outside your house”), and response options (eg, shortening “none of the time” to “none”) to clarify meaning, enhance readability, and aid in ease of translation and suitability for an electronic platform prior to testing in cognitive interviews.

Several final changes were made to the instructions and formatting of the questionnaire to facilitate display on an electronic device for data collection, with one item appearing per page. The resulting version 0.1 of the MPFID was used for the first round of cognitive testing.

### Cognitive interviews to test initial versions of the instrument

The characteristics of the 17 adults with migraine interviewed were consistent with patients who would participate in studies on preventative treatments for migraine (Table 1). Participants (82.3% female, mean age 39.5 ± 9.4 years) had a mean (SD) time since migraine diagnosis of 12.4 (11.3) years and a mean (SD) HIT-6 score of 62.4 (8.8) suggesting severe level of impact [27].

**Table 1:** Characteristics of Cognitive Interview Study Sample

	Overall *N* = 17
Age (years), mean (SD)	39.5 (9.4)
Sex (female), n (%)	14 (82.3)
Race (white), n (%)	11 (64.7)
Employment status, n (%)^a^	
Employed, full-time	10 (59)
Employed, part-time	3 (18)
Student	1 (6)
Unemployed	2 (12)
Disabled^b^	2 (12)
Highest level of education, n (%)	
Secondary/high school	2 (12)
Some college	6 (35)
College degree	7 (41)
Postgraduate degree	2 (12)
Overall HIT-6™ Score	62.4 (8.8)

^a^Not mutually exclusive ^b^Self-reported

With reference to the concepts covered in the instrument, most participants (10/16; 63%) felt that the MPFID was comprehensive with the items covering the range of their migraine experience (note: the denominator excludes one participant who was not probed on this topic). Six participants provided feedback related to how the MPFID could be more comprehensive; however, these suggestions were about specific symptoms rather than the impacts of the symptoms of migraine: two participants each suggested adding items on migraine symptoms, appetite, and sensitivity to light and sound; one participant suggested adding an item on nausea and sensitivity to smell. Since the intention of the instrument is to assess the impact of migraines on functioning, these items were not added. At the end of the first round, the instructions were clarified that respondents need to consider the **impact of all migraine symptoms** when responding to the items. Participant feedback also suggested that there was some overlap of concepts between items 4 (impact on ability to do activities than require concentration) and 5 (impact on ability to do activities that require thinking clearly). Because both concepts had been elicited during the CE interviews that were used as the basis for item generation, a decision was made to retain these two items and to examine them closely during item analyses in future validation studies to inform item reduction decisions. No revisions were made to the concepts covered in the instruments based on interviews.

Participants’ demonstrated a good understanding of the instructions, items, and response options. For example, when probed about the rationale for the choice of a specific response option related to the impact on the level of difficulty doing a task, interview participants were able to describe the intended meaning of the responses. For example: “Because when I have migraines it’s very tough for me to drive, I mean I’ve literally stopped and had to vomit, stop and go, stop and go, because I get really nauseated (006-226 –42 year old female)” “……., because, uh, when I have a migraine, when I had the last migraine, I still had to do activities or maybe I was in church and I needed to concentrate—I could, but if I’m having a migraine it’s very difficult to sit there and—I don’t even want to hear anyone talk, I just want to be in a dark room, lying down (006-226 –42 year old female)”.

Results of the interim analysis of the first round (*n* = 9) interviews suggested the need to refine the instructions to clarify the intent of the MPFID, to update items 2 (impact on ability to do activities outside of the home), 7 (impact on ability to interact with others), 9 (overall impact on ability to do usual activities), and 12 (impact on ability to get in and out of bed) to enhance specificity and clarity, and to revise item 6 (impact on ability to make yourself presentable) to include gender neutral language. The second round of interviews (*n* = 8) confirmed that the revisions were acceptable and that saturation was achieved. No new themes were identified after Round 1 that were determined to warrant changes to the content of the instrument. Item level feedback from the cognitive interviews is documented in Table 2.

**Table 2:** Summary of Item level Cognitive Interview Results

Item	Concept	Attribute measured^a^	Understanding n (%)^b^	Changes
Instructions			16 (94)	Two sentences added as a clarification to address patient feedback
Item 1	Impact on ability to do usual household chores	Difficulty	17 (100)	No changes
Item 2	Impact on ability to do activities outside of the home	Difficulty	17 (100)	Parenthetical phrase “For example shopping or doing errands” added to provide examples
Item 3	Impact on ability to keep routines/schedule	Difficulty	17 (100)	No changes
Item 4	Impact on ability to do Activities than require concentration	Difficulty	14 (82)	No changes
Item 5	Impact on ability to do activities that require thinking clearly	Difficulty	12 (71)	No changes
Item 6	Impact on ability to make yourself presentable	Difficulty	17 (100)	Item revised to clarify the concept as “getting ready for the day”
Item 7	Impact on ability to interact with others	Difficulty	17 (100)	Item and response options replaced by item on “avoiding interaction with other people”
Item 8	Needing to rest or lie down	Duration	17 (100)	No changes
Item 9	Overall Impact on ability to do usual activities	Difficulty	17 (100)	Response options “without any difficulty” to “unable to do” changed to “not difficult” to “extremely difficult”
Item 10	Impact on ability to move head	Duration	15 (88)	No changes
Item 11	Impact on ability to move body	Duration	15 (88)	No changes
Item 12	Impact on ability to get in and out of bed	Difficulty	17 (100)	Modified “get in and out of bed” to “get out of bed”
Item 13	Impact on ability to stand up	Difficulty	16 (94)	No changes
Item 14	Impact on ability to bend over	Difficulty	17 (100)	No changes
Item 15	Impact on ability to walk around inside home	Difficulty	17 (100)	No changes
Item 16	Impact on ability to walk at normal speed	Difficulty	17 (100)	No changes
Item 17	Impact on ability to do activities needing physical effort	Difficulty	17 (100)	No changes

^a^Response option type. ^b^ n (%) of subjects demonstrating that they understood the item as intended and were able to select a response to the item

## Discussion

The MPFID was developed and tested with adults with migraine using an iterative qualitative research process to confirm content validity [20, 28]. The methods followed the guidelines from the US FDA for the development of PRO instruments to support label claims [19, 20, 28]. The content of the instrument was based on insights from adults with migraine [19, 20, 28]. It is the first PRO instrument designed to collect data about the impact of migraine on everyday acts and tasks experienced on days with migraine as well as the days in-between migraines.

The MPFID was developed in response to the paucity of existing instruments for evaluating patient-reported concepts on the ***impact of migraine on physical functioning*** a key concept of interest for evaluating the benefits of interventions for the prevention of migraines [13]. Though existing instruments covered the impacts on everyday tasks, none of them included items about the impact on acts, that were identified both in the CE interviews that formed the basis of the development of the MPFID [13] but also identified by Leonardi et al. [12], when a broader framework following the ICF was used to explore impacts of migraine.

The MPFID was designed for completion as a daily diary to capture the impact on migraine days as well as the days in between migraines [4, 5, 13]. The MPFID can therefore be used to complement migraine diaries that usually include assessments about migraine symptoms. Though it has been recommended that daily headache diaries used in migraine trials include an item about the impact on quality of life [29], little evidence is available about the development and documentation about the content validity of these items per best practice methods [19, 20, 28]. Existing instruments that have some evidence of content validity (eg, MSQ [22] rely on longer recall periods (‘in past 4 weeks’) which can introduce recall bias, especially for an instrument that aims to capture the impacts that vary from day-to-day [30, 31]. While, both the HIT-6 and the MSQ aim to collect data about the frequency of experience, the MPFID was generated to reflect the experience of adults with migraine reported in the CE interviews [13] and include response options to evaluate the level of difficulty of functioning, which was also suggested by Leonardi et al. [12]. The results of the cognitive testing of the initial versions of the MPFID confirmed that these response choices resonated with interview participants.

Evidence to support the content validity of the instrument and documentation about a good understanding of the instrument in cognitive interviews (as required by guidance documents [20, 28]) of the instrument support its usability for evaluating functional outcomes of interventions for migraine in clinical research and practice, as suggested in guidelines for the management and evaluation of treatments for migraine [15–17].

Although the cognitive interview study was conducted on a small sample; it was designed using best practices suggested for instrument development, including testing in the target population and use of an. Further, the sample size was comparable to published suggestions of 5–15 participants per cognitive interview round [20, 28]. The cognitive interview sample was a clinic-based convenience sample that comprised mostly Caucasian and generally well educated patients; the use of the MPFID in a more generalized and diverse population will be examined as part of a prospective observational study. Next research steps include collecting data to inform item-reduction and test psychometric properties of the instrument to further confirm content validity, testing the usability of the MPFID on an electronic platform and, conducting translations for international use. The intent is to ensure that the final version of the MPFID is a clinically relevant and psychometrically sound measure of patients’ perceptions of functional impacts in adults with migraine for evaluating outcomes of preventive medication for migraine in clinical research and clinical practice.

## Conclusion

Instruments capturing migraine-specific impacts can enhance the evaluation of outcomes of interventions for migraine. We developed a new patient-reported outcome instrument based on qualitative data collected from adults with migraine to evaluate the impact of migraine on physical functioning. The results of the current study provide qualitative evidence supporting the content validity of the MPFID for evaluating outcomes of interventions for migraine.

## Additional file

Additional file 1: Table S1. Inclusion and Exclusion Criteria for Cognitive Interview Study. (DOCX 27 kb)

## Abbreviations

CE: concept elicitation
CM: chronic migraine
EM: episodic migraine
HIT-6™: Headache Impact Test™
MPFID: migraine physical function impact diary
PRO: patient-reported outcome
