# Supplementary material for: Development and exploration of the content validity of a patient-reported outcome measure to evaluate the impact of migraine- the migraine physical function impact diary (MPFID)
Source: Health Qual Life Outcomes. 2017 Nov 17;15:224. doi: 10.1186/s12955-017-0799-1 (PMC5693572; doi:10.1186/s12955-017-0799-1)
Supplement: Additional file 1: Table S1. — Inclusion and Exclusion Criteria for Cognitive Interview Study. (DOCX 27 kb) [file 12955_2017_799_MOESM1_ESM.docx]

Additional file 1: **Table S1**. Inclusion and Exclusion Criteria for Cognitive Interview Study

| **Inclusion Criteria:**  All of the following criteria had to be met: |
| --- |
| Adults between 18 and 60 years of age; |
| History of migraine headache (with or without aura) for ≥12 months prior to screening according to the IHS Classification ICHD-II (Headache Classification Committee of the International Headache Society 2004) based on medical records and/or patient self-report; |
| History of EM with a migraine frequency: ≥4 and ≤14 migraine headache days per month in each of the 3 months prior to screening. Subjects must experience a headache (i.e., migraine and non-migraine headache) frequency: <15 headache days per month (with >50% of the headache days being migraine days) in each of the 3 months prior to screening  OR  History of CM with a migraine frequency: ≥15 migraine headache days per month, with ≥8 migraine days per month in each of the 3 months prior to screening; |
| Able to read, speak, and understand English; |
| Willing and able to attend and participate in an interview, including discussion and completion of questionnaires; |
| Willing and able to provide informed consent to participate in research. |
| **Exclusion Criteria:**  Excluded if any of the following were present: |
| Previous participation in concept elicitation interview phase of AMG 334 protocol 20130172 Concept Elicitation Study; |
| Older than 50 years of age at migraine onset; |
| EM with more than 1 migraine with a duration of >72 hours in any month during the 3 months prior to screening; |
| CM with chronic migraine with continuous pain in which the subject did not experience any pain free periods (of any duration) during the 1 month prior to screening; |
| Unable to differentiate migraine from other headache, based on medical records or subject report; |
| History of cluster headache or hemiplegic migraine headache; |
| History or evidence of fibromyalgia or chronic pelvic pain syndrome; |
| Participation in any other clinical trial or receiving study drug or device within the previous 45 days from the date of screening for this study; |
| History of EM and has received botulinum toxin in the head and/or neck region within 6 months prior to screening; |
| Taking an opioid or butalbital-containing analgesic for any indication on greater than 2 days in any month during the 3 months prior to screening; |
| Cognitive impairment that would interfere with the subject’s ability to provide consent and participate in a cognitive interview; |
| History of major psychiatric disorder. Subjects with generalized anxiety disorder or major depressive disorder are permitted in the study if they are on no more than 1 medication for each disorder. Subjects must have been on a stable dose within the 3 months prior to screening and demonstrate clinical stability in the opinion of the investigator; |
| On anti-psychotic medication; |
| Employee and/or family and/or close relative of employee of the study site staff defined as spouse, parent, child, grandparent, or grandchild. |
